# Seismic background level (SBL) growth can reveal slowly developing long-term eruption precursors

**DOI:** 10.1038/s41598-023-32875-z

**Published:** 2023-04-12

**Authors:** Mie Ichihara, Takao Ohminato, Kostas I. Konstantinou, Kazuya Yamakawa, Atsushi Watanabe, Minoru Takeo

**Affiliations:** 1 Earthquake Research Institute, University of Tokyo, Yayoi 1-1-1, Bunkyo-ku, Tokyo, 113-0032 Japan; 2 Department of Earth Sciences, National Central University, Jhongli, 320 Taoyuan Taiwan; 3 Mount Fuji Research Institute Yamanashi Prefectural Government, 5597-1 Kenmarubi, Kamiyoshida, Fujiyoshida, Yamanashi 403-0005 Japan

**Keywords:** Seismology, Volcanology

## Abstract

The accelerating growth of seismic unrest before eruptions has been observed at many volcanoes and utilized for eruption forecasts. However, there are still many eruptions for which no precursory unrest has been identified, even at well-monitored volcanoes. The recent eruptions of Shinmoe-dake, Japan, have been another negative example of this kind. Here we present seismological evidence that the eruption preparation had been ongoing at the shallow depths beneath Shinmoe-dake for several months to a year. We investigated the seismic background level (SBL) of eleven-year data recorded around the volcano, including two stations about 1 km from the eruptive crater. We searched for persistent weak signals, focusing on low-amplitude time windows recorded during quiet nighttime. Then the spectra of daily background noise were classified by clustering analysis. The SBL analysis successfully revealed very weak precursory tremors from more than several months before the eruption, and residual tremors to the end of the eruptive period. The precursory signals grew acceleratory in a similar way as is assumed in the material failure forecast method applied to eruption forecasts. However, their growth was significantly slower and longer compared to other cases reported in the literature. Such slow and quiet eruption preparations would not be captured by conventional seismological methods. We expect that long-term SBL analyses on proximal seismic data will help detect early precursors, even at seismically quiet volcanoes, and will also help towards judging the end of an eruptive period.

## Introduction

An essential step toward the goal of forecasting volcanic eruptions is to capture the precursory signals based on the knowledge of the unrest patterns, either common to all volcanic systems or specific to each volcano, indicating magma migration to shallow depths^1–4^. Seismological methods are the most widely used monitoring techniques to achieve this^5–10^, among other effective techniques including the detection of geodetic^11,12^, degassing^13^, and thermal^14^ anomalies, as well as activation of minor or phreatic eruptions^15^. There are some successful cases where the detection of unrests and experiences from past eruptions led to effective early warning^16–18^ and quite a few cases where data analyses after eruptions revealed the possibility of eruption forecasts in retrospect^4,6^.

The significance of any unrest is not necessarily related to its magnitude. The advancements in instrumentation and data analyses have been uncovering hidden seismological signs of upcoming eruptions. The very-long-period (VLP, 2–100 s) signals get increasing attention as an eruption indicator^19–23^. Modern event-detection and relocation algorithms have revealed the detailed sequence of broadband seismic activity toward an eruption^24^. Vila et al.^25,26^ proposed to monitor the base level noise seismic spectrum (BLNSS) to detect early signals of volcanic unrest and found that BLNSS gradually increased over 10 months before ash eruptive episodes at Llaima volcano, Chile, in April, 2003^25^. Analyzing non-volcanic persistent noise using seismic interferometry^27,28^ or permutation entropy^29,30^ could extract subtle underground changes prior to eruptions. These studies raised a hope that we may observe seismological precursors for any eruption with adequate monitoring systems and analytical techniques. On the other hand, there are still many major eruptions for which no precursory unrest was identified or, the unrest was observed within a very short time span^3,4^. Such a situation may happen even at well-monitored volcanoes like the 2014 eruption at Kuchinoerabujima, Japan^31^ and the 2019 paroxysms at Stromboli, Italy^32^.

**Figure 1:**
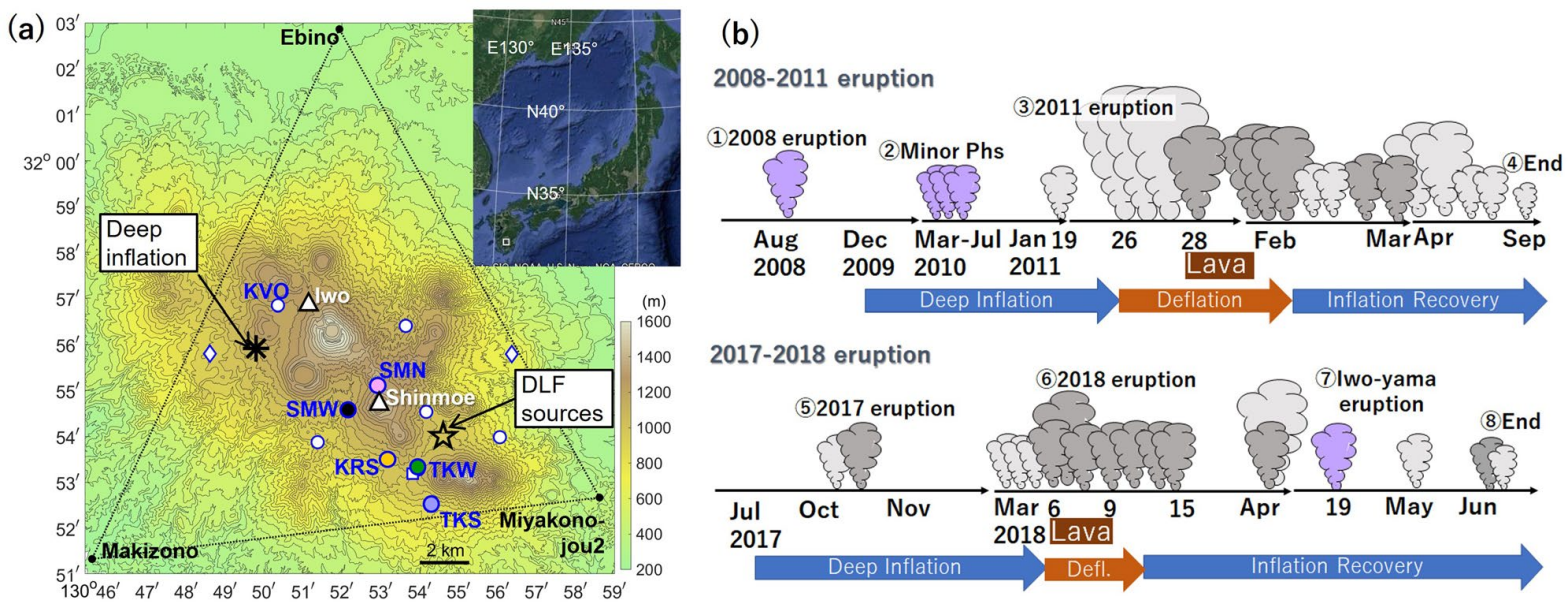
Overview of the monitoring system and the eruption sequences at Shinmoe-dake. (**a**) A map of Kirishima volcanic group in southwestern Japan (the white square in the inset: Goole Earth, Data SIO. NOAA.U.S. Navy NGA GEBCO). The white triangles indicate Shinmoe-dake and Iwo-yama. The colored circles are seismic stations used in this study, and white markers show other seismic stations. The vertices of the black triangle are the GEONET stations calculating the areal strain^43^, which represents the deep inflation beneath the asterisk^40^. The star indicates the epicenters of deep low-frequency earthquakes, whose rate correlated with the inflation at the asterisk^44^. (**b**) A schematic representation of Shinmoe-dake eruption sequences^34,41^. ① The August 2008 phreatic eruption, ② minor phreatic eruptions, ③ the main phase of the 2011 eruption, ④ the end of the 2011 eruption, ⑤ the 2017 eruption, ⑥the main phase of the 2018 eruption, ⑦ the April 2018 phreatic eruption at Iwo-yama, and ⑧ the end of the 2018 eruption. The purple, light-gray, and dark-gray clouds indicate phreatic, ash-forming, and vulcanian eruptions, respectively.

**Figure 2:**
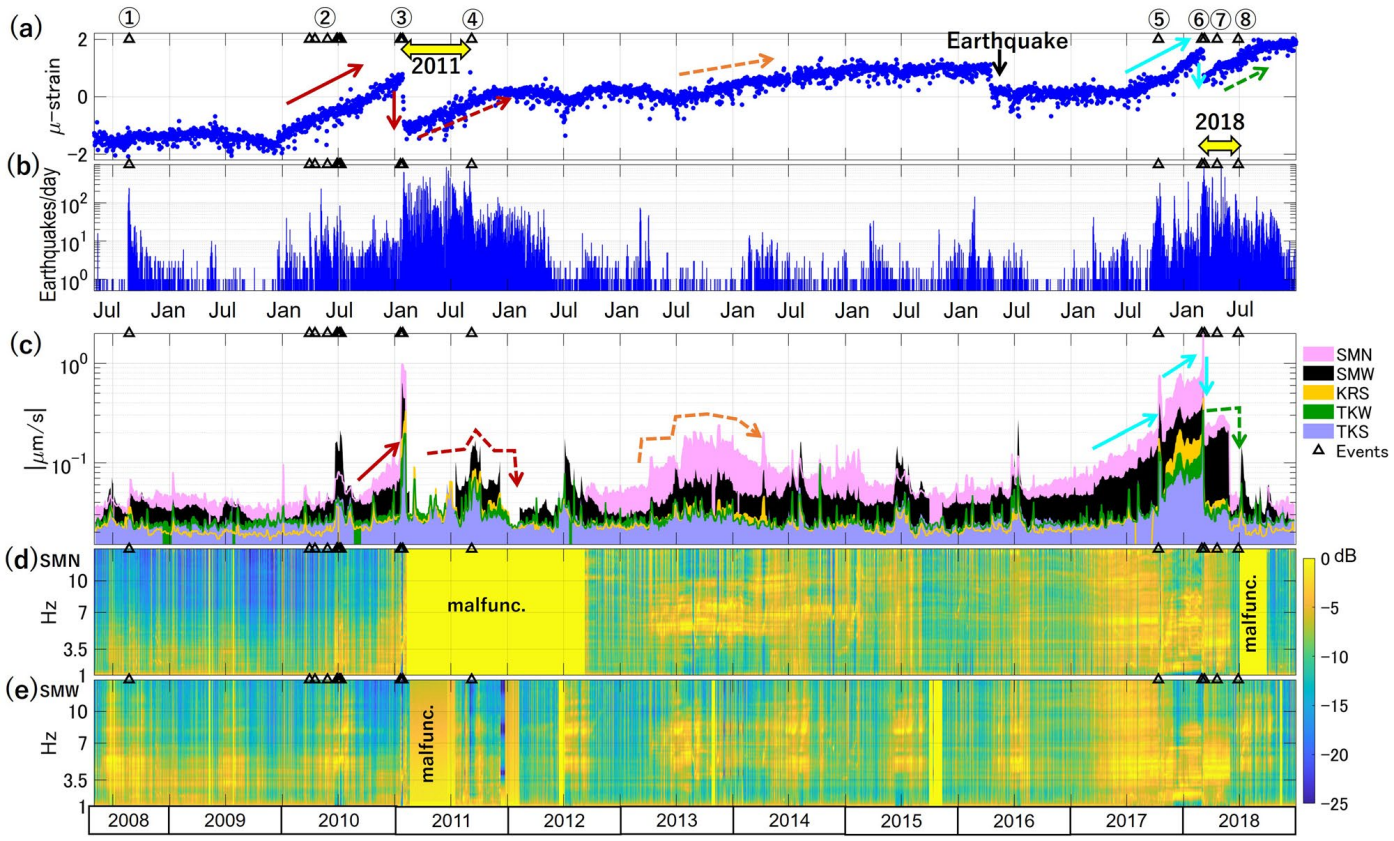
The variations of SBL compared to other monitoring data. (**a**) The areal strain (in micro-strain) indicating the deep inflation^43^. (**b**) Daily counts of shallow earthquakes determined by JMA beneath Shinmoe-dake. The count does not include tremor or very-long-period events. (**c**) Logarithmic plots of weekly smoothed SBLs at the five stations indicated by the corresponding colors in Fig. 1a. The area below each SBL value is color-filled for better visibility of the SBL ratio to the next stations. (**d**) and (**e**) The daily SBL spectra, ${P}_{SBL}$, at SMN and SMW, respectively. The black triangles and circled numbers mark the events represented in Fig. 1b.

The recent eruptions of Shinmoe-dake, an active cone of the Kirishima volcanic group in southern Kyushu, Japan (Fig. 1), added another negative example of this kind. Shinmoe-dake became active following a phreatic eruption in August 2008. Subsequently, two magmatic eruptions occurred in 2011 and 2017–2018, erupting $50-70 \times {10}^{9}$ kg^33^ and $\sim 30\times {10}^{9}$ kg^34,35^ respectively. The 2011 event was the first major magmatic eruption of Shinmoe-dake since 1716–1717 producing $\sim 200\times {10}^{9}$ kg of tephra^36,37^, though eruptions involving small magmatic activity in 1822 ($<{10}^{9}$ kg of tephra) and 1959 (mainly phreatic eruption generating $\sim 9\times {10}^{9}$ kg of tephra) have been documented^36,37^, as well as minor phreatic eruptions (1991–1992, 2008–2010)^33,36^. Based on these records, Nakada et al.^38^ defined Shinmoe-dake as an example of less-frequent magmatic activity. As a consequence, the volcano before the 2011 eruption could be characterized as a closed system^39^ for which the precursory unrest should in general be more apparent^4^. A dense monitoring system had been operated at Kirishima, including two seismic stations about 1 km from Shinmoe-dake crater (Fig. 1a). The geodetic observation revealed a year-long inflation (the red upward arrow in Fig. 2a), whose source was about 10 km deep beneath the asterisk in Fig. 1a^40^. Because there were multiple active edifices closer to the inflation source, scientists had not considered its linkage to Shinmoe-dake until they observed its deflation upon the eruption (the red downward arrow in Fig. 2a)^41^. This inflation-deflation pattern was observed also during the 2018 eruption (the blue arrows in Fig. 2a). The seismic event rate beneath Shinmoe-dake attained relatively high values (~ 10 events/day) (Fig. 2b)^41,42^. Yamada et al.^42^ reported an increase of the number of low-frequency (LF) events, but not their magnitudes, before the eruptions. However, the seismicity exhibited no significant acceleration to the eruptions. For the last 10 years, scientists have been searching for geophysical signs indicating magma migration to shallow depths beneath Shinmoe-dake. Seismic interferometry revealed a slight decrease in seismic velocity beneath the crater in less than a month before the 2011 eruption, but no decrease before the 2018 eruption^43^. Kurihara et al.^44,45^ found that the event rate of deep low-frequency (DLF) earthquakes deeper than 17 km in the southeast (see star in Fig. 1a) showed a good correlation with the deep inflation in the northwest prior to the eruptions. Yet, any shallow precursors that were significant or common to both eruptions have not been identified to date^42,43^.

Here we report the first seismological evidence that the preparation of eruptions at Shinmoe-dake had been ongoing at the shallow depth for several months to 1 year. We investigated the seismic background level (SBL) that is similar to the BLNSS^25,26^, using only the selected silent periods of seismic data during the nighttime of each day. The SBL analysis of the eleven-year record allowed us to recognize weak precursory continuous tremor that was slowly growing prior to the 2011 and 2017–2018 eruptions, and whose growth accelerated toward the eruptions. We expect that such long-term weak seismic precursors may exist before more apparent signals are initiated prior to other eruptions as well, even at seismically quiet volcanoes.

## Results

Figure 1a shows the permanent seismic stations used in this study by colored markers while other stations are indicated by open markers. Some stations had short-period seismometers, and some had broadband seismometers (Fig. S1.1). All seismic stations recorded continuously with a sampling rate of 100 Hz. We analyzed the frequency range from 1 to 15 Hz. This paper mainly presents the SBL in 3.5–7 Hz because the variation of interest was the most visible. Although many reported volcanic tremors have dominant frequencies around 1 Hz^46^, frequency bands above several Hz are also informative^47,48^.

We analyze the data from May 1, 2008 to December 31, 2018. All stations except SMW were operated with the same instruments individually in the key periods discussed in this study (Fig. S1.1). At SMW station, we used data from short-period (1 Hz) seismometers before August 1, 2010, and a broadband seismometer afterward. We confirmed that the SBL growth before the 2011 eruption was captured by both sensors consistently (Fig. S1.2). When we discuss temporal variation at the individual stations, we do not correct for the site amplification effects in this study.

### Temporal SBL variation

We calculated daily SBL in 3.5–7 Hz band and smoothed them by week to reduce the short-term effect (See Methods). Figure 2c presents the smoothed SBL of the five stations (colored circles in Fig. 1a). We observe characteristic variations indicated with arrows, which are prominent and correlated at the two stations closest to the crater (SMN and SMW) as well as temporally related to the deep inflation-deflation behaviors (arrows with corresponding colors and styles in Fig. 2a). These variations are compared with the eruptive events described in the next paragraph. We also noticed the elevated SBL every summer at all the stations, which is the most evident at SMW. Figure 2d and e present the stacked and normalized power spectra in the quiet time windows every night, which we refer to as ${P}_{SBL}$ (see “Methods”), at SMN and SMW, respectively. We see the spectral structures change with the characteristic variations of SBL.

We observe growth of SBL before the 2011 eruption (the red arrow in Fig. 2c) and before the 2017 and 2018 eruptions (the light-blue arrows in Fig. 2c) simultaneously at SMN and SMW. The growth is visible also at more distant stations after July 2017 (KRS station malfunctioned). Then, SBL becomes apparently larger than usual at all the stations, growing from the 2017 eruption to the 2018. In this period, the persistent oscillation characterizing SBL dominates the daytime human noise and can be identified as ‘continuous tremor’^49^. SBL decreases after the main phase of the 2018 eruption but does not return to the normal level until the end of May. The SBL increase and slight inflation of the deep source are observed in 2013 and 2014 without an eruption (the dashed orange arrows in Fig. 2a and c). The elevated SBL in this period has different features from those prior to the 2011 and 2017 eruptions. The increase is stepwise, the SBL ratio of SMN to SMW is apparently larger (Fig. 2c), and ${P}_{SBL}$ is poor in low-frequency components and has varying peaks (Fig. 2d and e).

We also note the SBL variations toward the end of eruptive periods. Although the deep inflation resumed after the co-eruption drop, it did not accompany SBL growth. After the main phase of the 2018 eruption, SBL stayed at the level prior to the 2017 eruption but abruptly went back to a lower level at the end of May 2018, while the recovery inflation continued for several months (the dashed-green arrows in Fig. 2a and c). The 2017–2018 eruption ceased with two minor events in June 2018 (Fig. 1b). In the 2011 eruption, the close stations had operational problems after the main phase. Nevertheless, the other stations suggest that SBL remained relatively high, exhibited a peak at the end of the 2011 eruptive period, and declined at the end of the year, when the recovery inflation stopped (the dashed red arrows in Fig. 2a and c).

**Figure 3:**
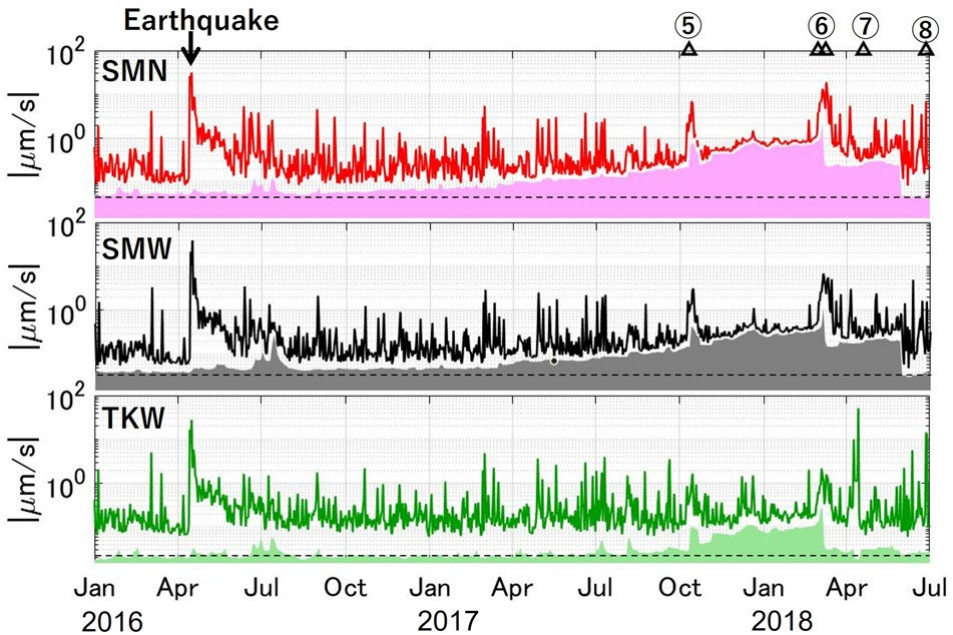
Comparison between RSEM (lines) and SBL at SMN, SMW, and TKW (patched areas) from 2016 to 2018. The horizontal dashed lines are put as a reference of the normal level. Figure S4 shows the comparison at all the stations during the whole period.

All the above-mentioned features are not apparent in daily RSEM or RSAM (Figs. 3 and S4). On the other hand, the RSEM is more sensitive to non-volcanic signals, including the 2016 Kumamoto-Oita earthquake sequence and its aftershocks, whose hypocenters are located 60–170 km away from Shinmoe-dake^50^.

### Clustering analysis results

To quantify the spectral features described above (Fig. 2d and e), we performed clustering classification^51,52^ of ${P}_{SBL}$ (see “Methods”). The current clustering method emphasizes the similarity in overall trend of spectra rather than local features like positions and shapes of spectral peaks. Besides, the number of clusters is arbitrary. Below we give similar names and colors to clusters that appear in similar periods.

**Figure 4:**
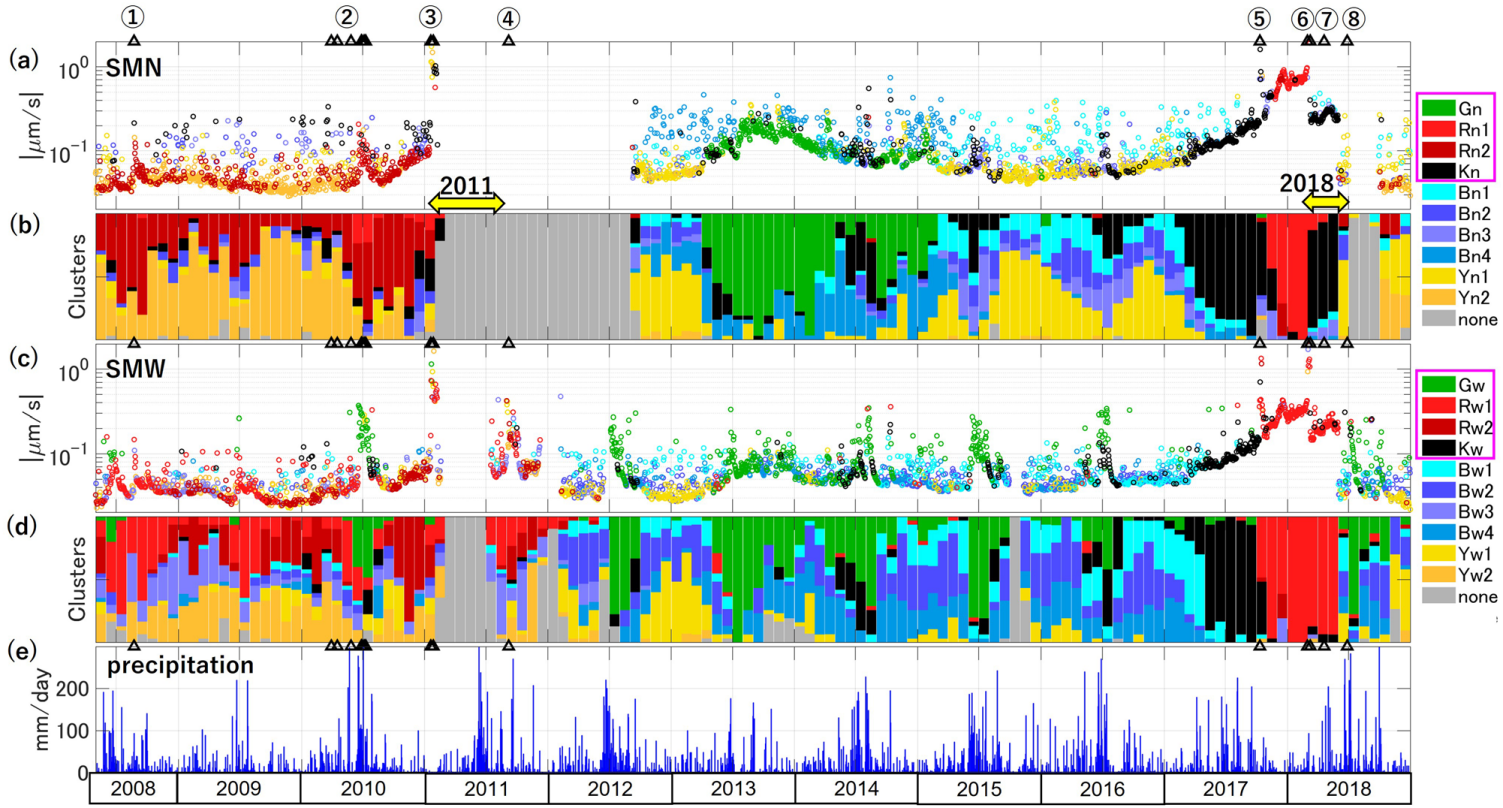
The clustering analysis results at SMN (ab) and at SMW (cd) for the daily SBL spectra, ${P}_{SBL}$, shown in Fig. 2d and e, respectively. (**a**,**c**) The smaller value of the SBLs in the night window and the next morning window is selected as the SBL of each day and is plotted with a circle of the color corresponding to its spectral cluster as in the legend, (**b**,**d**) The monthly fractions of days belonging to each cluster are shown by bars of the same color. The clusters of current interest are enclosed by the magenta rectangles in the legends. The gray clusters represent data with problems. (**e**) The daily precipitation data recorded near KVO station in Fig. 1a. The black triangles and circled numbers are the same as in Fig. 2.

The daily SBL values belonging to different clusters are distinguished by the colors in Fig. 4a and c, and monthly fractions of each cluster are shown in Fig. 4b and d. The characteristic variations of the smoothed SBL are represented mainly by green, red, and black clusters (enclosed by the magenta rectangles in the legends of Fig. 4). The blue and yellow clusters exhibit short-term day-by-day fluctuation and appear only in the quiet periods (SBL is generally small, and the volcanic activity is also low). Thus we regard them unrelated to the long-term SBL growth of current interest. Some components of the red and black clusters also exhibit short-term fluctuation in 2008–2010, which we discuss later.

The green cluster at SMW (Gw) mainly appears in the summer when SBL values are high, and correlates with the local precipitation (Fig. 4e). Although the specific mechanism for this is not known, the increased water flow may generate higher levels of noise, especially at station SMW that is located near a running river. However, the Gw cluster is also observed in 2013–2014 independently of the precipitation. At SMN, the green cluster (Gn) exclusively appears in the 2013–2014 period. Therefore, the high values of SBL in this period are regarded as unrelated to precipitation. Another transient increase in SBL at SMW in September 2011 (around ④) does not include the green clusters nor correlates with precipitation. We can infer that the increase is not caused by precipitation either, although the stations were not fully functioning in this period.

The red clusters dominate as SBL increases prior to the 2011 eruption and between the 2017 and 2018 eruptions at SMN (Rn1 and Rn2) and SMW (Rw1 and Rw2). The increase of SBL prior to the 2017 eruption is dominated by black clusters (Kn at SMN and Kw at SMW). Both Kn and Kw increase with high precipitation as well, indicating that the SBL that grows prior to the 2017 eruption has some similarity to the precipitation noise. Also, the black clusters sparsely appear from 2008 to the 2011 eruption and generate scattered SBL values. However, the SBL behavior prior to the 2017 eruption is distinct because of the large values, the growth that is independent of the precipitation, and additional spectral features that have not been distinguished by the current clustering method but are visually apparent (Fig. 2d). The red and black clusters also constitute the SBL in the decaying period of the 2018 eruption from March to May 2018. From the beginning to the 2011 eruption, the red clusters are always present at both stations, some of which generate daily scattered variations. This may be partly because the different sensors before and after the 2011 eruptions (Fig. S1.1) may have affected the clustering analysis. On the other hand, it is also possible that the red clusters reflect the fact that the volcano was always active in that period with several phreatic eruptions.

### Signal locations

**Figure 5:**
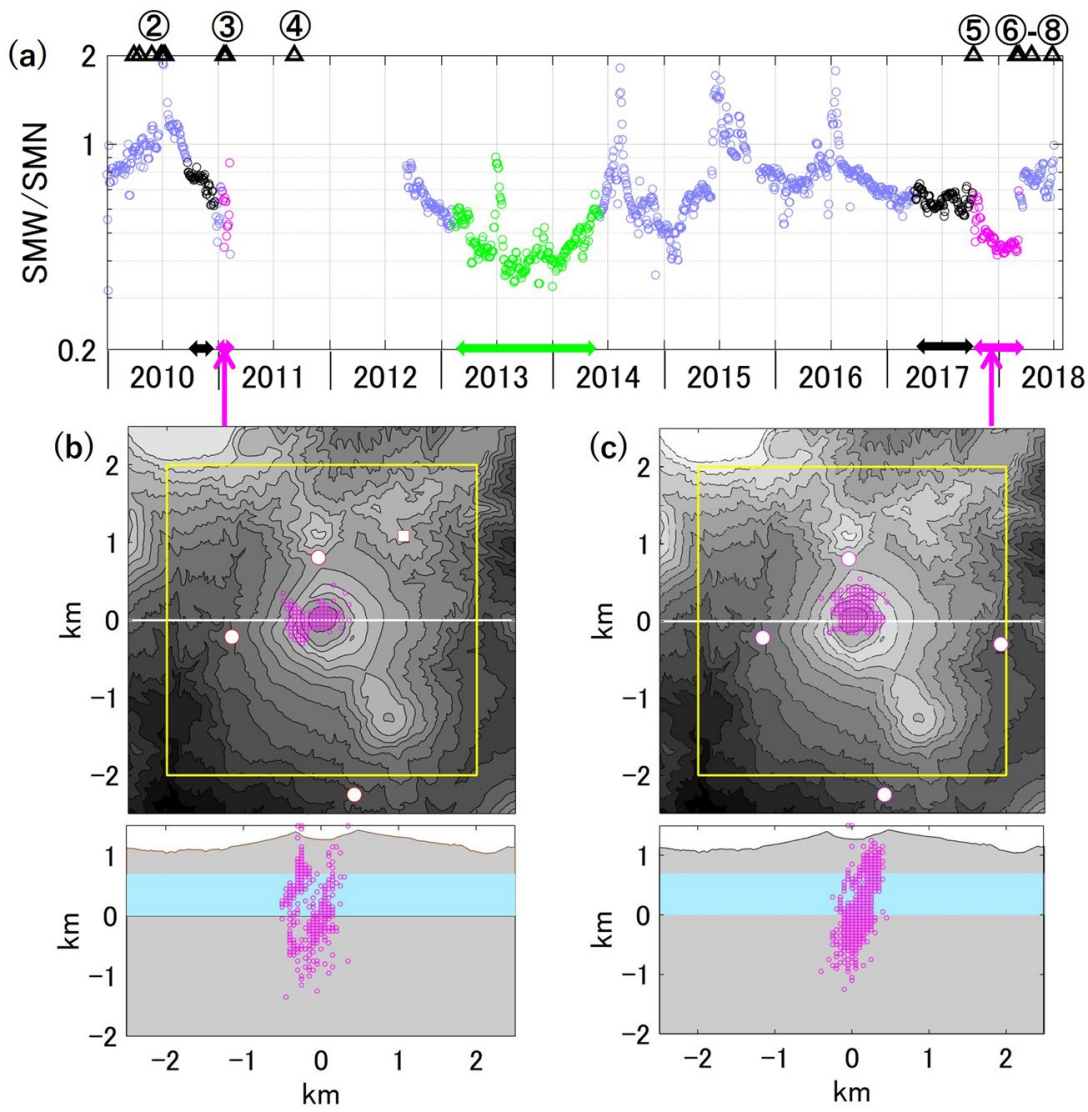
(**a**) The ratio of weekly smoothed SBLs at SMW to SMN. Note that the vertical axis is logarithmic. Black indicates precursory periods (September 20–December 20, 2010 and April 1 –October 10, 2017), green indicates the non-eruptive SBL increase in 2013–2014 (February 10, 2013–June 1, 2014), and pink indicates volcanic tremor periods analyzed by Ichihara and Matsumoto^47^ (January 13–February 7, 2011) and in this study (October 10, 2017–March 9, 2018). The black triangles and circled numbers are the same as in Fig. 2. (**b**,**c**) The tremor sources are presented for the 2011 eruption in (**b**)^47^ and for the 2017–2018 eruption in (**c**). The upper panel shows the topographic map centered at the Shnmoe-dake crater. The yellow frame encloses the area we searched for the sources (see Ichihara and Matsumoto^47^). The cross section along the white line is displayed in the bottom panel. The vertical axis is the elevation above sea level, and light-blue area indicates the water-table depth^56^.

The SBL remains high from the 2017 eruption to the 2018 eruption (from ⑤ to ⑥ in Fig. 2c), obscuring the daytime human noise. We estimate the source location of the signals that characterize the high SBL using the same method as in the previous study for the 2011 eruption (the amplitude-based source location in the frequency band 3.5–7 Hz)^47^. The tremor sources for the 2011 eruption (Fig. 5b)^47^ and the 2017–2018 eruptions (Figs. 5c) are distributed over a similar region beneath Shinmoe-dake.

In relation to these confirmed tremors, we investigate the SBL ratio between SMN and SMW stations, where the elevated levels of SBL prior to the eruptions is apparent. Figure 5a reveals that the SMW/SMN ratio is larger in the precursory periods (black arrows) than during the tremor periods (pink arrows) and during the elevated SBLs in 2013–2014 without subsequent eruptions (a green arrow). The logarithmic plot of SBL (Fig. 2c) also indicates that the ratios of SBLs at SMN and SMW to the other stations are larger in the precursory periods than in the other periods. From these ratios, we infer that the precursory SBL sources are shallower and more to the west than the tremors. To determine the source locations, we need to investigate the SBL distribution with more stations around the sources, which will be the goal of a future study.

## Discussion

The accelerating growth of seismic measures, $\dot{\Omega }$ (e.g. tremor amplitude, BLNSS, energy rate, and event rates), is a typical feature of eruption precursors, which has been utilized referring to the material failure forecast method (FFM)^53^. FFM represents the growth of $\dot{\Omega }$ with time $t$ by $\mathrm{d}\dot{\Omega }/dt=A{\dot{\Omega }}^{\alpha }$, where $A$ and $\alpha$ are constants. It has empirically been shown that most of the volcanic precursors are fitted with $\alpha =2$, so that $1/\dot{\Omega }$ linearly decreases with time^4–9,54^. Then, the trends were fitted by

1 $$\begin{array}{c}\frac{1}{\dot{\Omega }}=a\left({t}_{s}-t\right),\end{array}$$

where ${t}_{s}$ is the time when $\dot{\Omega }$ becomes infinity and can be regarded as the upper limit for the time of failure, ${t}_{f}$. Because the determination of ${t}_{f}$ itself is controversial, FFM is mainly used to estimate ${t}_{s}$^5,6,54^.

**Figure 6:**
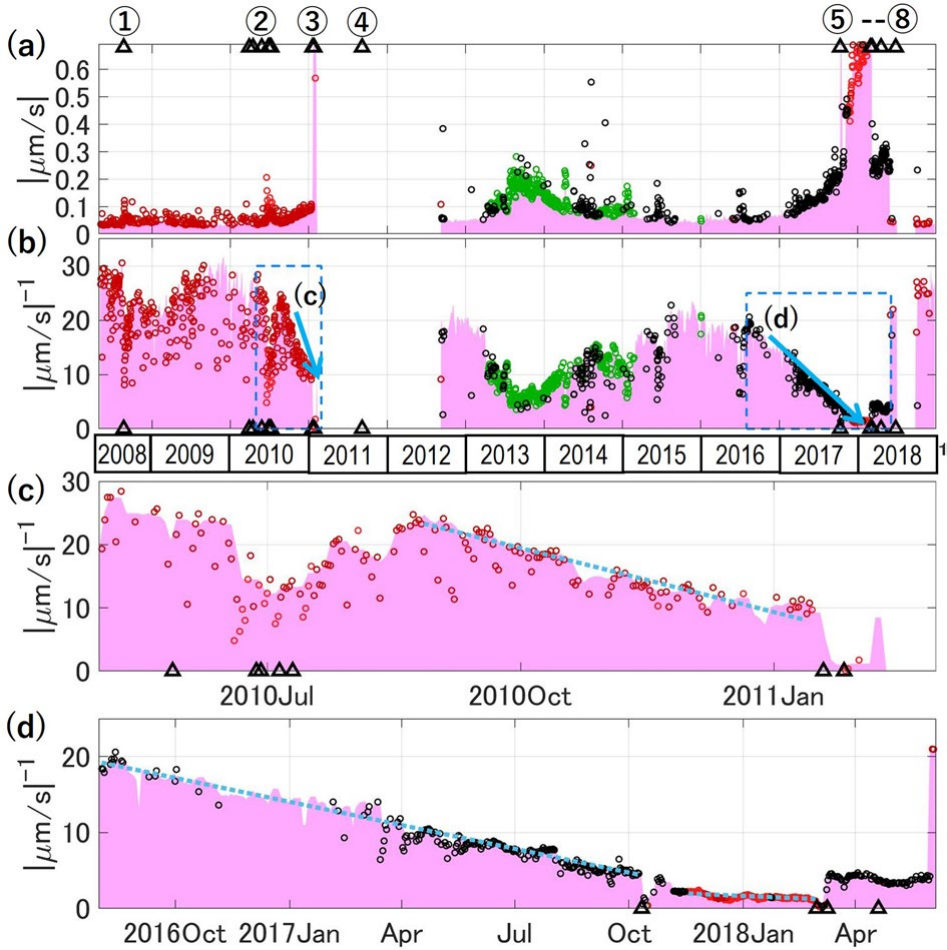
Application of FFM to SBL. (**a**) The daily SBL at SMN belonging to the clusters related to volcanic activity (Gn, Rn1, Rn2, and Kn). The colors are the same as in Fig. 5a. The light-magenta area shows the weekly smoothed SBL at SMN as in Fig. 2c. (**b**–**d**). The inverse of the data in (**a**), and the magnifications of the rectangles, (**c**) and (**d**). They exhibit linear slopes down toward the 2011 eruption (**c**) and the 2017 and 2018 eruptions (**d**). The dotted lines show the fitting of the weekly smoothed SBL by Eq. (1) with the parameters listed in Table 1.

Figure 6 shows the smoothed SBL (Fig. 2c) and the daily SBL belonging to the relevant clusters (red before the 2011 eruption and red, green, and black afterward) in a linear scale (a). Their inverse (b) exhibits the linear downslope of 1/SBL prior to the 2011 (Fig. 6c), 2017, and 2018 eruptions (Fig. 6d). They are individually fitted by Eq. (1) with $[{t}_{s}\left(\mathrm{day}\right), a\left(\mathrm{\mu m}{\mathrm{s}}^{-1}/\mathrm{day}\right)]$ listed in Table 1. The durations (100–300 days) of these downslopes are significantly longer than those previously reported for accelerating precursors fitted to FFM, which had a duration in the order of 10 days or less^4,5,26,54^. The SBL acceleration may signify that some catastrophic processes had been slowly developing at the shallow depths of Shinmoe-dake for many months before the eruptions.

**Table 1:** Parameters for fitting the SBL growth prior to eruptions by FFM.

Fitting period	${t}_{s}\left(\mathrm{day}\right)$	$a\left(\mathrm{\mu m}{\mathrm{s}}^{-1}/\mathrm{day}\right)$	Residual (%)
Aug 25,2010–Jan 13, 2011	Mar 27, 2011	0.11	7.3
Aug 1, 2016–Oct 9, 2017	Feb 17, 2018	0.034	7.2
Nov 17, 2017–Feb 28, 2018	Aug 30, 2018	0.0069	13

The elevated SBLs in 2013 and 2014 without subsequent eruptions exhibit temporal and spatial patterns different from the precursory SBL. In this period, the daily SBL spectra (${P}_{SBL}$) had distinct features with significant power in 5–10 Hz (see Gn and Gw clusters in Fig. S5). Also at the same period, Kirishima volcanic group shows several unrest signs, including the deep inflation (Fig. 2a) and increases in seismicity and volcanic tremor beneath various cones (JMA, 2020). More case studies are necessary to understand the variability and mechanisms of eruptive and non-eruptive SBL elevation.

Recently, Jolly et al.^55^ found slow evolution of very weak continuous tremors associated with ‘Silent’ dome emplacement at White Island, New Zealand, and interpreted them to be generated by the interaction of the hydrothermal system and slowly propagating magma. The central to northwestern region of Kirishima volcanic group is also known for abundant underground water at shallow depth^56,57^. The signal found in SBL are generated at shallow depth, so that it might have a similar hydrothermal mechanism. We expect that long-term SBL analyses on proximal seismic data will help towards the extraction of such weak and slowly evolving precursory signals at the volcanoes where conventional methods fail to recognize unrest. It is also noted that the SBL monitoring is potentially useful when one needs to judge the end of an eruption period. Further studies are necessary for clarifying the source locations and mechanisms of the SBL noise.

## Conclusion

The seismic background level (SBL) was evaluated, focusing on low-amplitude periods of seismic data recorded during quiet nighttime. It revealed precursory signals of Shinmoe-dake eruptions. The signals were evident mainly at the closest stations that were around 1 km from the eruptive crater. Our analysis indicated that the preparation of eruptions at Shinmoe-dake had been ongoing right beneath Shinmoe-dake for many months before the eruptions. This finding will change the view of eruption preparation timescales, including the development of magma pathway from the deep reservoir to the crater. SBL exhibited accelerating increases before the eruptions, which was comparable with the prediction of FFM. On the other hand, the volcanic signals measured as SBL were significantly weaker, longer, and slower in their acceleration than the previously reported seismic precursors for eruptions at other volcanoes. The slow and quiet development in the shallow magma plumbing system toward an eruption might occur at volcanoes with developed hydrothermal systems, though such volcanos may also have strong precursory seismicity. The signatures observed with SBL were not apparent in conventional seismological parameters, including event rate and seismic amplitude measurements. We expect that long-term SBL analyses on proximal seismic data will help detect early precursors, even at seismically quiet volcanoes. Such an analysis could potentially be useful for the purpose of judging the end of an eruptive period.

## Methods

### Calculation of SBL and SBL spectra

We calculated the power spectral densities, ${P}_{f}(t,f)$, from the three components at each station in a time window of 10.24 s sliding every 10 s, where $t$ is the central time of the window, and $f$ is the frequency from 1 to 15 Hz at 0.1-Hz intervals. Integrating ${P}_{f}$ from 3.5 to 7 Hz, we obtained the seismic power in the band, $E(t)$. There was daily variation of $E(t)$ mainly due to human noise, which is low during night (6 pm-6am, see Fig. S2). We investigated the distribution of $\sqrt{E(t)}$ for the nighttime data (morning window, 00:00–06:00 JST, and night window, 18:00–24:00 JST) and employed the lowest 20-% value (the 20-th percentile) of $\sqrt{E(t)}$ in each of the morning and night windows as the daily SBL (two points per day). Changing the threshold from 20 to 5% did not lead to significant differences in the results (Fig. S3). We did not use the lowest value to avoid outliers, caused by missing data. We found short-term (daily to weekly) fluctuations, mainly caused by weather effects. To suppress them, we smoothed the daily SBL by taking the 20-th percentile in a seven-day window (14 points) sliding every 2 days. Alternatively, the daily average of $E(t)$ or $\sqrt{E(t)}$ is comparable with the daily value of real-time seismic energy measurement (RSEM)^10,58^ or, more commonly, the real-time seismic amplitude measurement (RSAM)^7^ in the target frequency band. Note that RSEM is defined as $E(t)$ or $\sqrt{E(t)}$ by different authors in the literature. Here we employ the latter definition to make RSEM directly comparable to SBL (Figs. 3 and S4).

We also calculated the stacked and normalized SBL spectrum of each day, $d$, which is referred to as ${P}_{SBL}(d,f)$. The specific procedures are as follows. First, we selected time windows of which $E(t)$ values were between 5 and 20% from the lowest in each nighttime (from 18:00 on day $d-1$ to 6:00 on $d$). Second, we averaged ${P}_{f}(t,f)$ of the selected time windows by day and normalized it by the maximum value at 1–15 Hz.

### Clustering analysis

Clustering analysis is becoming a popular tool in volcanology^59–61^. We applied a clustering analysis to characterize the daily SBL spectra of day $d$, ${P}_{SBL}(d,f)$. The clustering analysis was performed individually for SMN and SMW stations. The protocol was the same as Sect.  “Clustering analysis” of Yamakawa et al. (2022)^61^ applied to infrasound source directions. Here, we used ${P}_{SBL}(d,f)$ as the input parameters.

Firstly, we defined the squared distance, ${L}_{mn}^{2}$, between ${P}_{SBL}$ of days ${d}_{m}$ and ${d}_{n}$ as

$${L}_{mn}^{2}={\int }_{1.5}^{12}{\left[{\mathrm{log}}_{10}{P}_{SBL}({d}_{m},f)-{\mathrm{log}}_{10}{P}_{SBL}({d}_{n},f)\right]}^{2}df.$$

We selected the frequency band of 1.5–12 Hz after several trials in 1–15 Hz to capture both volcanic and non-volcanic features we see in Fig. 2d and e. We obtained a distance matrix, ${\varvec{L}}$, whose elements are the inter-element distances, ${L}_{mn}$ (${L}_{nn}=0$ and ${L}_{mn}={L}_{nm}$). Secondly, we combined the matrix elements into clusters recursively following the general agglomerative hierarchical clustering algorithm^52^. In every step, we updated the distance matrix with fewer elements representing the inter-cluster distances, employing the Lance-Williams recurrence formula^62,63^ based on Ward’s method^51^. Note that Ward's method is known to be less affected by outliers. The result was visualized in the form of a dendrogram that shows the similarity relations among the different elements. Then we manually defined the boundary of the clusters according to the structure of the dendrogram confirmed by viewing the spectra in each cluster (Fig. S5).

## Supplementary Information

Supplementary Information.

## Data Availability

The precipitation data and seismic event catalog are provided by the Japan Meteorological Agency (JMA) (http://www.jma.go.jp), and the GNSS data are by Geospatial Information Authority of Japan (https://www.gsi.go.jp). The data to reproduce Figs. 1a, 2, 3, 4, 5 and 6 and figures in Supporting Information are open through zenodo (https://doi.org/10.5281/zenodo.7779735) as Matlab fig files. The raw seismometer data at Kirishima are available under the joint usage of data and records of the Earthquake Research Institute of the University of Tokyo (https://www.eri.u-tokyo.ac.jp/en/joint-usage-top/).
