# Supplementary material for: Seismic background level (SBL) growth can reveal slowly developing long-term eruption precursors
Source: Sci Rep. 2023 Apr 12;13:5954. doi: 10.1038/s41598-023-32875-z (PMC10097692; doi:10.1038/s41598-023-32875-z)
Supplement: Supplementary file 1 — Supplementary Information. [file 41598_2023_32875_MOESM1_ESM.pdf]

Supplementary method 1
Seismic station specifications

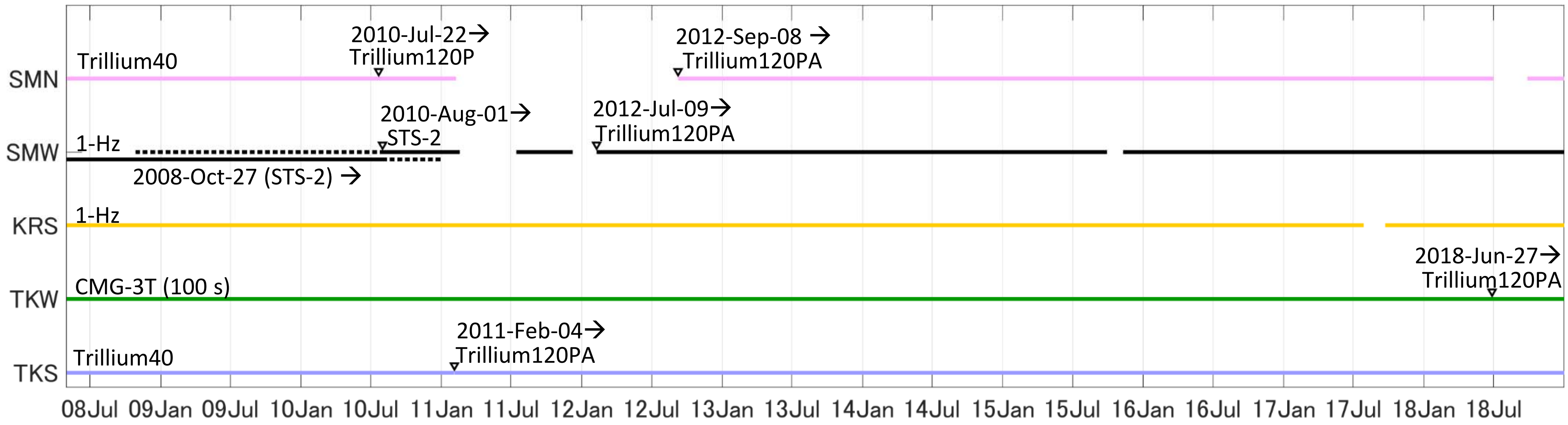

Fig. S1.1.
Summary of seismometers operated at the stations used in this study. The inverted triangles indicate the points that the seismometers used in this study are changed. The gaps of the lines indicate malfunctioning periods.

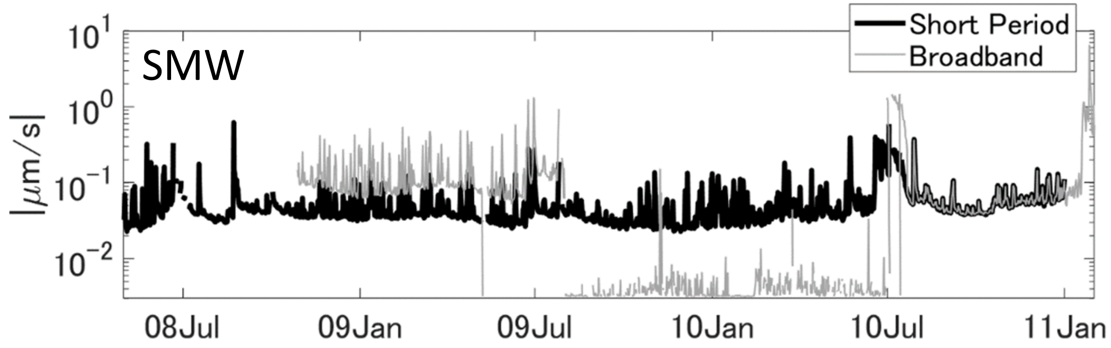

Fig. S1.2.
Comparison of SBL at SMW between the pre-installed short period seismometer and the broadband seismometer operated from October 27, 2008. The latter was unstable at the beginning of operation, but both agreed after August 2010. We use the data from the short period before August 2010 and the broadband seismometer afterward.

Supplementary method 2
Daily variation of seismic power and calculation of SBL

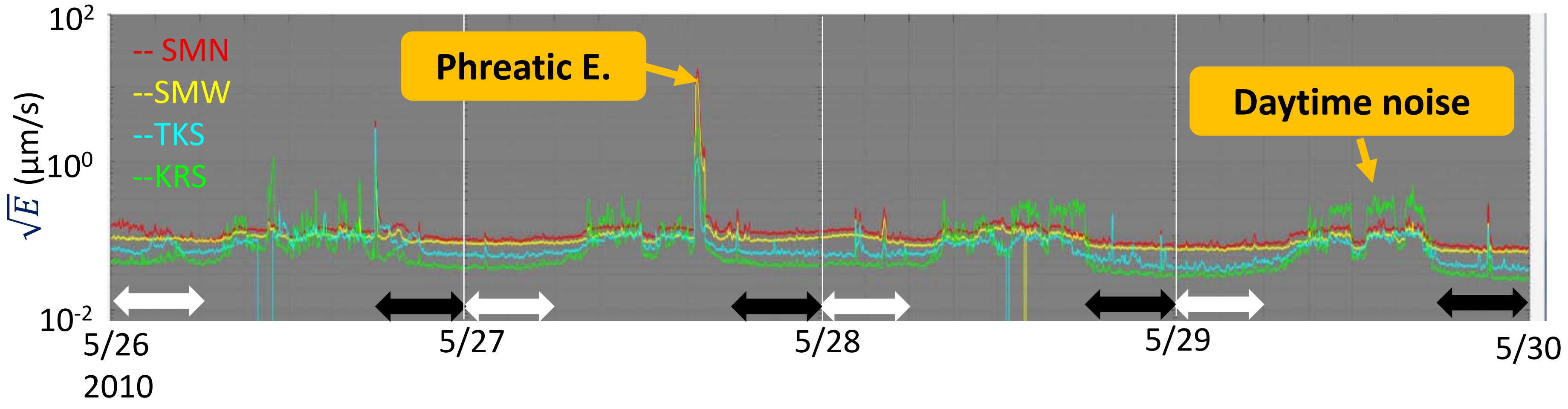

Fig. S2. Variations of seismic RMS amplitudes for four days. White and black arrows indicate the time windows (morning window, 00:00 – 06:00, and night window, 18:00 – 24:00 JST) that we used to calculate SBL.

Supplementary method 3
Calculation of SBL using a different percentile

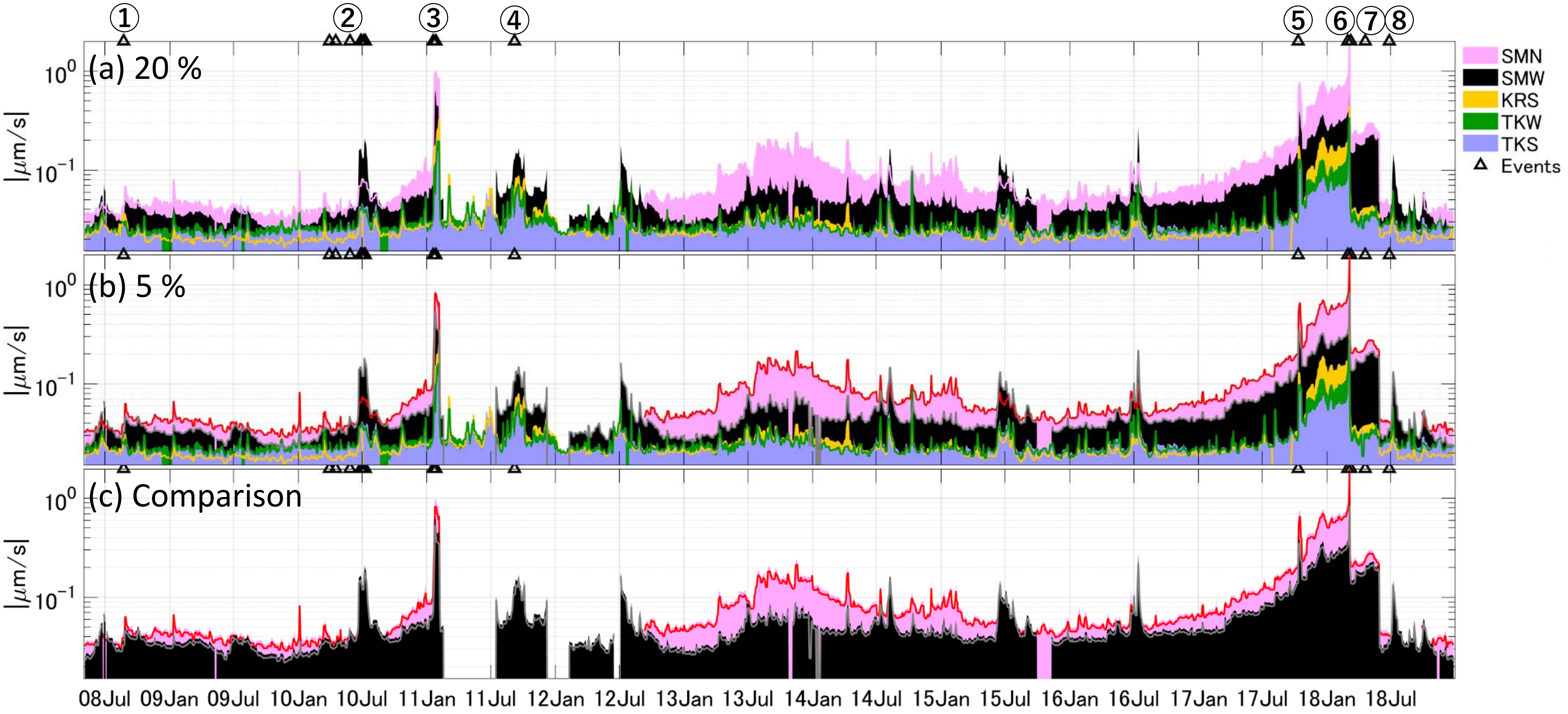

Fig. S3. The smoothed SBL using the daily lowest 20-% amplitude (a) (the same plot as Fig. 2(c) in the text) and the lowest 5-% values (b). The results at stations SMN and SMW are compared in (c). The light-magenta and black colored areas are from (a) while the red and gray lines are from (b).

Supplementary method 4
Comparison of SBL with conventional daily RSEM

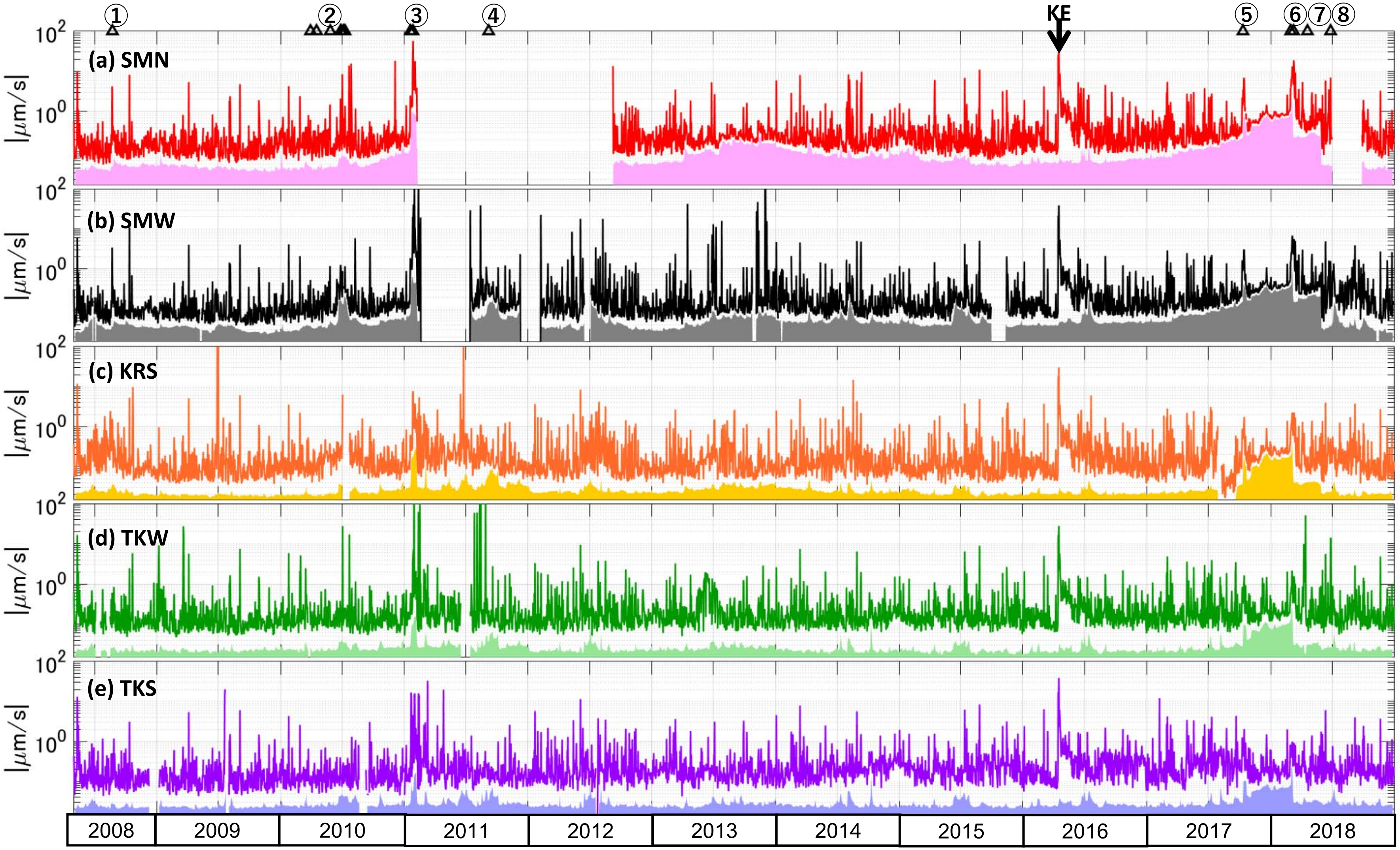

Fig. S4. The daily RSEM (lines) and the weekly smoothed daily SBL (colored patches), both in 3.5－7 Hz, are compared at the five stations used in this study. The black triangles and circles numbers mark the events represented in Fig. 1b. The onset of the Kumamoto earthquake on April 14, 2016, is indicated with an arrow (KE).

Supplementary method 5
Spectra classification by the clustering analysis

We performed a clustering analysis of the stacked and normalized daily SBL spectra, $P_{SBL}(d, f)$, and constructed dendrogram, individually at SMN (Fig. S5a) and SMW (Fig. S5b). According to the dendrograms and spectral features, we defined eleven clusters at SMN and twelve at SMW. The names and colors of the clusters are used in the text. We focus on the green, black, and two red clusters at each station.

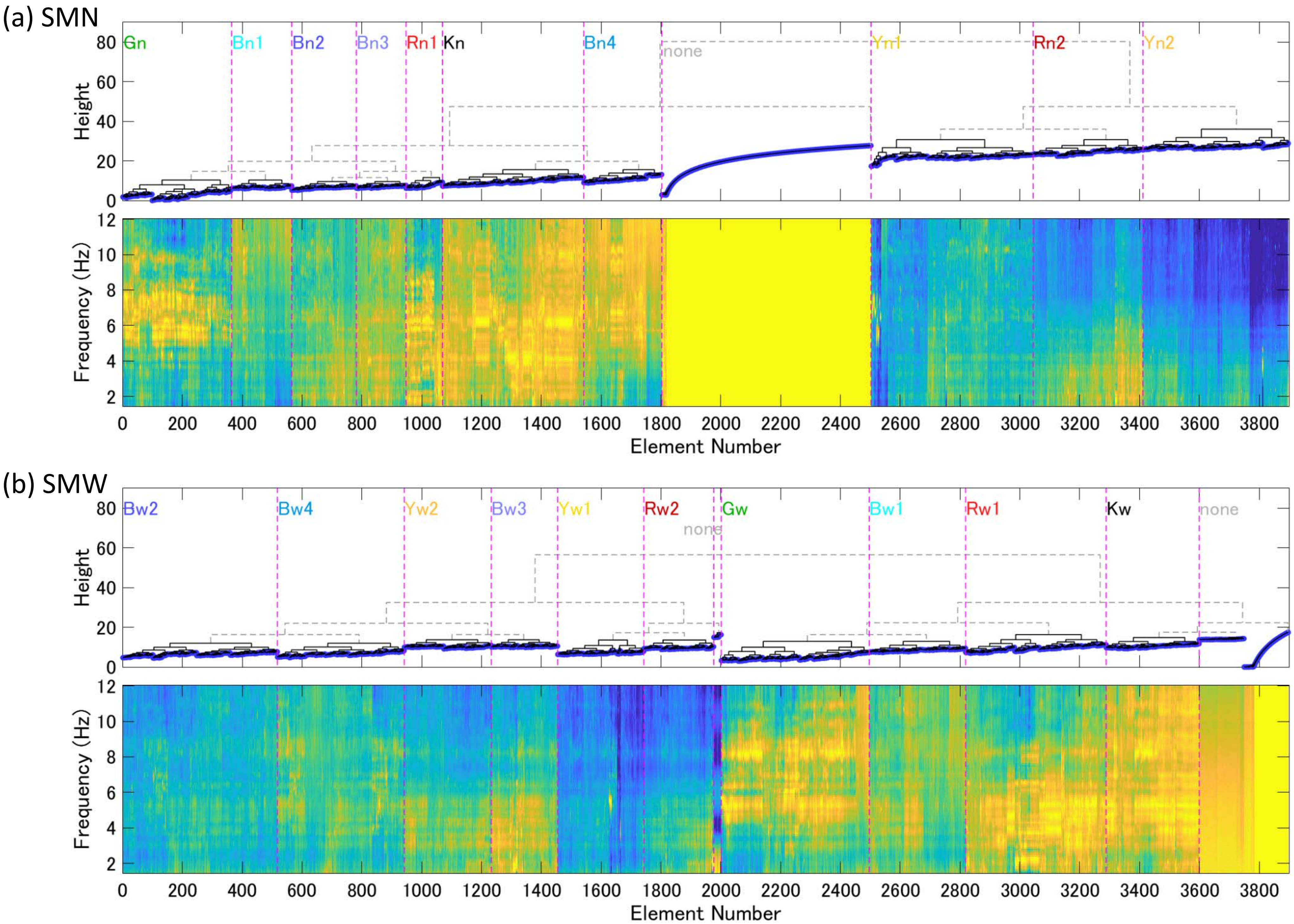

Fig. S5. Dendrograms and sorted daily SBL spectra, P_{SBL} obtained by the clustering analysis. The dashed magenta lines separate clusters.
